# Elective induction of labor: A prospective observational study

**DOI:** 10.1371/journal.pone.0208098

**Published:** 2018-11-29

**Authors:** Malin Dögl, Pål Romundstad, Line Dahlgaard Berntzen, Oliv Camilla Fremgaarden, Katrine Kirial, Anne Molne Kjøllesdal, Benedicte S. Nygaard, Line Robberstad, Thorbjørn Steen, Christian Tappert, Cecilie Fredvik Torkildsen, Magdalena R. Vaernesbranden, Alexander Vietheer, Runa Heimstad

**Affiliations:** 1 Department of Obstetrics and Gynecology, St. Olav's Hospital, University Hospital of Trondheim, Trondheim, Norway; 2 Department of Clinical and Molecular Medicine, Faculty of Medicine and Health Sciences, Norwegian University of Science and Technology, Trondheim, Norway; 3 Department of Public Health, Faculty of Medicine, Norwegian University of Science and Technology, Trondheim, Norway; 4 Department of Obstetrics, Oslo University Hospital, Oslo, Norway; 5 Department of Gynecology and Obstetrics, Innlandet Hospital Trust, Elverum, Norway; 6 Department of Gynecology and Obstetrics, Stavanger University Hospital, Stavanger, Norway; 7 Department of Gynecology and Obstetrics, Vestre Viken Hospital Trust, Drammen, Norway; 8 Department of Obstetrics and Gynecology, Sørlandet Hospital, Kristiansand, Norway; 9 Department of Obstetrics and Gynecology, Ålesund Hospital, Møre and Romsdal Hospital Trust, Ålesund, Norway; 10 Department of Gynecology and Obstetrics, Haugesund Hospital, Haugesund, Norway; 11 Department of Gynecology and Obstetrics, Østfold Hospital Trust, Norway; 12 Department of Obstetrics and Gynecology, Haukeland University Hospital, Bergen, Norway; Monash University, AUSTRALIA

## Abstract

The aim of the present study was to assess indications for induction and describe the characteristics and delivery outcome in medical compared to non-medical/elective inductions. During a three-month period, 1663 term inductions were registered in 24 delivery units in Norway. Inclusion criteria were singleton pregnancies with cephalic presentation at gestational age 37^+0^ and beyond. Indications, pre-induction Bishop scores, mode of delivery and adverse maternal and fetal outcomes were registered, and compared between the medically indicated and elective induction groups. Ten percent of the inductions were elective, and the four most common indications were maternal request (35%), a previous negative delivery experience or difficult obstetric history (19%), maternal fatigue/tiredness (17%) and anxiety (15%). Nearly half of these inductions were performed at 39^+0^–40^+6^ weeks. There were fewer nulliparous women in the elective compared to the medically indicated induction group, 16% vs. 52% (p<0.05). The cesarean section rate in the elective induction group was 14% and 17% in the medically indicated group (14% vs. 17%, OR = 0.8, 95% CI 0.5–1.3). We found that one in ten inductions in Norway is performed without a strict medical indication and 86% of these inductions resulted in vaginal delivery.

## Introduction

Induction of labor is common and the rates are increasing in most western countries[1, 2]. According to the European Perinatal Health Report from 2010, 15 of 25 European countries had an induction rate above 20%[3]. In Norway, one in eight women had their labor induced in 2003. Ten years later, this had increased to one in five according to the Medical Birth Registry of Norway[4]. This trend is probably multifactorial, where better induction methods and more liberal indications, such as the rising prevalence of induction for maternal requests, are major contributing factors [5, 6]. An improved ability to plan the timing of the delivery by the patient, her family and the physician is a common reason for induction, as is induction of labor for logistic reasons[6]. Implemented patient empowerment may also contribute. Elective inductions, defined as inductions of labor in the absence of medical or obstetrical indications, are common and contribute to the overall increasing induction rate[5, 7–9]. The prevalence of elective induction in Norway is not known[10].

The aim of this study was to assess and describe factors associated with elective induction including indications of labor induction, and to compare maternal and fetal outcomes for medically indicated and elective inductions.

## Materials and methods

All delivery units in Norway inducing labor were invited to participate in this prospective observational study. Twenty-four of 39 hospitals accepted participation. The participating hospitals were Ålesund Hospital, Bærum Hospital, Drammen Hospital, Elverum Hospital, Haugesund Hospital, Haukeland University Hospital, Kirkenes Hospital, Kongsvinger Hospital, Kristiansund Hospital, Levanger Hospital, Lillehammer Hospital, Mo i Rana Hospital, Molde Hospital, Namsos Hospital, Oslo University Hospital, Østfold Hospital Trust, Ringerike Hospital, Sandnessjøen Hospital, Stavanger University Hospital, St. Olavs Hospital, Sørlandet Hospital Kristiansand, Telemark Hospital, Tønsberg Hospital and Vesterålen Hospital. The included delivery units are located in every region and accounted for 73% of all deliveries in Norway in 2013. The study period was January 2013—March 2013. Two hospitals included patients during January and February 2013 only. The study population was restricted to induced labors at term in a singleton pregnancy in cephalic presentation at a gestational age 37^+0^ and beyond. A web-based case report form was used with a unique login for each hospital.

Demographic data, obstetric history, Bishop scores prior to induction and detailed information about the induction procedure were registered. Indications were predefined and listed in a multiple-choice format. It was requested to state a main indication in case of combined or several indications. Information about labor and delivery, including classification of cardiotocography (CTG) during the first and second stage of labor, the mode of delivery, indications for assisted vaginal delivery or cesarean section (CS), anesthetics and analgesic methods, maternal blood loss, birthweight, Apgar scores at 1, 5 and 10 minutes and admission to neonatal intensive care unit (NICU) were registered. Duration of hospitalization and maternal and fetal diagnoses were registered at discharge.

All the inductions were started before the regular uterine contractions started, and cases with augmentation solely were excluded. The indications for induction were grouped as medical or elective. Medical indication included post-term pregnancy, hypertensive disorders in pregnancy, intrauterine growth restriction (IUGR) or small-for-gestational age (SGA), oligohydramnios, pre-labor rupture of membranes, pre-existing or gestational diabetes mellitus, fetal macrosomia, unstable lie, polyhydramnios, maternal and fetal morbidity. Maternal morbidity included non-obstetric medical conditions. Fetal morbidity mostly included cases with non-reassuring fetal status.

In Norway, pregnancies are dated by the 18–19 week ultrasound scan. According to the Norwegian Directorate of Health guidelines, induction for post-term pregnancy should start no later than day 294 (42^+0^ weeks). The induction is recommended to be initiated earlier if a situation of oligohydramnios, SGA/IUGR, discrepancy of more than 14 days between last menstrual period estimated date of delivery (EDD) and ultrasound-based EDD, pre-gestational body mass index (BMI) > 30kg/m^2^, or age > 35 years for nulliparous women is present at an antenatal examination one week past the ultrasound-based EDD. Oligohydramnios is defined as amniotic fluid index ≤5cm and/or single deepest pocket ≤2cm. Elective induction is defined as induction without strict medical maternal or fetal benefit for delivery compared with continuation of pregnancy.

Hemorrhage was defined as blood loss >500ml for vaginal delivery and >1000ml at cesarean section. Cesarean section was categorized into two groups relating to degree of urgency. Category 1 is the highest degree of urgency with immediate threat to the life of the woman or fetus. Category 2 includes all other CS. CTG was classified as normal, intermediary and abnormal as defined by national guidelines[11]. The study was approved by the Regional Committee for Medical and Health Research Ethics, Central Norway (approval no 2012/1240).The ethics committee waived the need for participant consent. Categorical variables were compared using the chi-squared test, and continuous variables with the *t*-test. For multivariable analyses we used logistic regression and adjusted for maternal age, pre-gestational BMI and parity to assess potential confounding. Both unadjusted and adjusted results were reported with odds ratio (OR) and 95% CI. A value of *p*< 0.05 was considered significant.

## Results

During the study period, 1928 women had their labors induced. Of these, 1663 (86%) met the inclusion criteria. The characteristics of the population are shown in Table 1. The groups were similar with respect to educational status, BMI and Bishop score at induction. Parity was lower in the medically induced compared to the elective induction group, 52% and 16% nulliparous women, respectively (*p*<0.001). There was a statistic significant difference in mean age between the groups. The women in the medically indicated group were slightly younger with a mean age of 30.3 compared to 31.6 in the elective induction group (*p* = 0.007), but this is not considered to be clinically significant.

**Table 1:** Characteristics of the population.

	Elective induction^a^ N = 158		Medical indication for induction of labor^b^ N = 1505	
	n	%	n	%
Maternal age (years)				
<25	24	15	233	16
25–34	77	49	924	61
≥35	57	36	348	23
Educational level				
9 years	4	3	30	2
12 years	61	41	557	41
>12 years	83	56	782	57
Nulliparous woman	25	16	788	52
Pregestational BMI				
<18.5	4	3	43	3
18.5–24.99	79	53	763	54
25–29.99	37	25	360	26
≥30	29	20	248	18
Gestational age at induction (weeks+days)				
<39^+0^	41	26	301	20
39^+0^–40^+6^	77	49	437	29
≥41^+0^	40	25	767	51
Bishop score				
≤5	120	78	1271	87
>5	34	22	196	13

^a^Missing data (Educational level n = 10, Pregestational BMI n = 9, BMI at induction n = 18, Bishop score n = 4)

^b^Missing data (Educational level n = 136, Pregestational BMI n = 91, BMI at induction n = 196, Bishop score n = 38)

Overall, 39% of the inductions were initiated with Foley catheter. There was no difference in Foley catheter use for cervical ripening in the two groups. In the medically indicated group, 70% were induced with prostaglandins (misoprostol vaginal tablet, dinoprostone endocervical gel, vaginal tablet or gel), whereas this was 60% among the electively induced (*p =* 0.01). Oxytocin infusion, either as augmentation or induction was used in 56% of labors with no difference between the two groups.

Ten percent (n = 158) were elective and 90% (n = 1555) were medically induced. Post-term pregnancy and prelabor rupture of membranes were the most common primary indications (Table 2). Among the elective inductions, the four most common indications were maternal request (35%), history of a difficult delivery experience/obstetric history (19%), maternal fatigue/tiredness in pregnancy (17%) and anxiety (15%). The remaining indications were pelvic and/or low back pain and induction to avoid CS on maternal request.

**Table 2:** Main indication for induction of labor (N = 1663).

	N	%
Post-term	496	29.8
Prelabour rupture of membranes	297	17.9
Elective	158	9.5
Preeclampsia	141	8.5
Oligohydramnios	134	8.1
Maternal morbidity	128	7.7
IUGR/SGA	90	5.4
Diabetes mellitus	64	3.8
Fetal morbidity	55	3.3
Hypertension	50	3.0
Fetal macrosomia	27	1.6
Unstable lie	12	0.7
Polyhydramnios	11	0.7

The mean gestational age at delivery was higher in the medically indicated group (40^+4^ and 40^+0^ weeks, respectively, *p*<0.001). Table 3 presents the maternal and fetal outcomes. CTG-use during first stage of labor, CS rates, hemorrhage and admission to NICU were similar for the two groups, also when adjusted for age, parity and pre-gestational BMI. The incidence of Apgar score <7 at 5 minutes did not differ substantially between the groups (0.7% in the elective vs. 1.2% in the medical induction group). Abnormal CTG in the second stage of labor and operative vaginal deliveries occurred less often, while vaginal deliveries occurred more often in the elective induction group compared to the women with a medical indication. However, the differences between the groups disappeared after adjusting for age, parity and pre-gestational BMI, with parity as the only variable showing a difference.

**Table 3:** Logistic regression analyses of maternal and fetal outcomes for elective induction of labor vs. medical induction of labor.

		Elective induction N = 158	Medical indication for induction of labor N = 1505				
		n (%)	n (%)	Crude		Adjusted^a^	
				OR	95%CI	OR	95%CI
CTG in first stage of labor							
Abnormal		5 (3.6)	88 (6.7)	0.52	0.21–1.30	0.66	0.26–1.70
CTG in second stage of labor							
Abnormal		29 (23.2)	380 (32.1)	0.64	0.42–0.99	0.80	0.51–1.25
Delivery ≤ 24h		73 (50.7)	765 (55.7)	0.82	0.58–1.15	0.61	0.42–0.87
Mode of delivery							
Vaginal delivery		114 (78.6)	934 (67.8)	1.75	1.16–2.64	0.97	0.62–1.51
Operative vaginal delivery		11 (7.6)	211 (15.3)	0.45	0.24–0.85	0.84	0.43–1.62
Cesarean section		20 (13.8)	233 (16.9)	0.79	0.48–1.29	1.19	0.71–2.01
Epidural analgesia		75 (53.6)	710 (52.8)	1.03	0.73–1.47	1.84	1.26–2.68
Hemorrhage		18 (12.6)	197 (14.4)	0.86	0.51–1.44	1.02	0.60–1.73
Apgar score 5min < 7		1 (0.7)	16 (1.2)	0.59	0.08–4.49	0.59	0.07–4.77
Admission to NICU		8 (5.7)	92 (6.8)	0.83	0.39–1.74	0.76	0.35–1.63

^a^Adjusted for age, parity and pregestational BMI

In the medically indicated group, there was a higher proportion of deliveries within 24 hours after induction and less use of epidural analgesia when adjusting for age, parity and pre-gestational BMI. In additional analyses, we adjusted for Bishop score and gestational age, but this did not change the results. The mean birthweight was 3702 g in the elective compared to 3600 g in the medically indicated group (*p* = 0.03). The proportion with third- and fourth-degree perineal tears was 1.8% among all induced labors, but no substantial differences were found between the groups. The mean time for hospital stay was similar in the two groups with 4.5 and 4.1 days in the elective and medically induced, respectively.

## Discussion

Elective induction is the third most common indication for induction of labor in term pregnancies, and one in ten induced labors were performed without medical indication in Norway. In addition, it was noteworthy that the overall cesarean section rate in all these induced labors was low.

In the USA, 16% of term deliveries were induced without medical indication [9]. Coulm et al. found that 13.9% of the induced labors were elective in France [5]. Even if the rate of elective inductions in Norway seem to be slightly lower than in other western countries, the increase in elective inductions is still of concern.

In accordance with other studies, the main factor associated with elective induction compared to medically indicated induction was parity, with a markedly higher rate of multiparous women in the elective group [5, 8, 12, 13]. A substantial amount of elective inductions in the present study was due to a negative delivery experience and/or obstetric history. The contribution from midwives and obstetricians to facilitate a positive birth experience during the first labor is of utmost importance, as this may influence the subsequent pregnancy and delivery. Educational level and BMI have also been reported to be associated with elective induction [7, 13, 14], but this was not found in our study.

In a Norwegian questionnaire study, all delivery units were asked at what gestational age elective inductions were done. None replied that this occurred before 37 weeks and “close to estimated date of delivery” was most common [10]. This is in accordance with our findings where half of all elective inductions were performed at a gestational age between 39 and 41 weeks. We found that the odds for epidural anesthesia were higher in the elective induction compared to the medical indicated group when adjusted for parity, maternal age and pre-gestational BMI. The same applied after adjusting for Bishop score and gestational age at induction. This may be due to the number of women with a history of negative delivery experience and anxiety in the elective induction group.

Currently there is no consensus as to whether elective induction should be offered to women requesting induction without medical indication. Several studies have shown an increased rate of cesarean section related to elective inductions, especially studies with spontaneous onset of labor as comparison group [15–18]. This will not reflect a real-life situation since the obstetrician’s and the woman’s options are induction or continuation of pregnancy. Continuation of the pregnancy will increase the chance of spontaneous onset of labor, but also increase the risk of developing pregnancy complications such as preeclampsia, macrosomia and oligohydramnios. Other studies have compared elective induction with all women delivered at a higher gestational age. These studies show similar or decreased cesarean section rates [13, 19, 20]. In a randomized controlled study including 161 nulliparous women with an unfavorable cervix, elective induction after 39^+0^ weeks of gestation was compared to expectant management. There was not a statistically significant increase in the cesarean section rate for elective induction compared to expectant management, but the authors still consider the difference to have a clinically relevant value (31% vs. 18%) [21].

In the present study we compared the elective inductions with medically indicated inductions. Based on the pathological pregnancies in the medical induction group, we expected a higher cesarean section rate in the medically indicated induction group. We found low cesarean section rates in both groups, 14% in the elective induction group and 17% in the medically indicated induction group. In a cohort with 115 528 deliveries, a lower cesarean section rate was found after elective induction compared to medically indicated induction, but spontaneous onset of labor was associated with the lowest cesarean section rate [12]. In contrast, a retrospective cohort study of 13 971 term deliveries by Boud et al described a cesarean section rate of 27% in the medically indicated and 29% in the elective induction groups [7].

The sample size is small in our study, which is a limitation. Furthermore, the small sample size limited subgroup analyses. Another limitation of present study is the missing data that could have affected the results. In register studies or retrospective studies, the indication of labor induction has been assumed to be elective in the absence of an indication in the registers or records [17, 18, 20]. The strength of present study is the use of prospective pre-specified indications for induction of labor. This ensured that all elective inductions were indeed done without a medical indication. Since the participating delivery units represent three quarters of all delivery units in Norway, we assume that the data in the present study are representative for the population of women having their labor induced in Norway during the study period.

In present study, we found a high rate of vaginal delivery after electively induced labor. Despite these results, women opting for elective induction should be informed of the risk of unsuccessful induction.

## Abbreviations

CTG: cardiotocography
CS: cesarean section
NICU: neonatal intensive care unit
IUGR: intrauterine growth restriction
SGA: small for gestational age
EDD: estimated date of delivery
BMI: body mass index
